# Impact of the COVID-19 pandemic on ophthalmic specialist training in Poland

**DOI:** 10.1371/journal.pone.0257876

**Published:** 2021-09-24

**Authors:** Joanna Konopińska, Iwona Obuchowska, Łukasz Lisowski, Natalia Dub, Diana Anna Dmuchowska, Marek Rękas

**Affiliations:** 1 Department of Ophthalmology, Medical University of Bialystok, Bialystok, Poland; 2 Department of Ophthalmology, Military Institute of Medicine, Warsaw, Poland; University of Zambia, ZAMBIA

## Abstract

The aim of this study was to evaluate the impact of the coronavirus disease 2019 (COVID-19) pandemic on ophthalmology residency training in Poland. An anonymous self-administered online survey involving polish ophthalmology residents was conducted between February 15 and 28, 2021. Of the 126 residents who completed the survey, 88.9% (n = 112) and 89.7% (n = 113) felt that the COVID-19 pandemic had negatively impacted their surgical training and overall training programs, respectively. Trainees providing care to patients with COVID-19 and female trainees indicated a greater negative impact of the pandemic on the implementation of the specialization program (p = 0.008 and p = 0.015, respectively) and on the acquisition of practical skills (p = 0.014 and p = 0.021, respectively). Approximately 94% (n = 118) of the residents surveyed participated in virtual training during the pandemic, and 99.2% (n = 124) positively assessed the content and usefulness of online teaching in everyday clinical practice. The most common platforms used for online meetings were Zoom (62.7%, n = 79) and Microsoft Teams (50.8%, n = 64). Approximately 79% (n = 99) of residents believed that, after the pandemic is over, at least some of the training courses and conferences should be conducted online. In addition, 13.5% (n = 17) of residents reported that they would like to undergo training and specialization courses in virtual form only after the pandemic is over. In summary, the present findings indicate that the COVID-19 pandemic negatively impacted the implementation of the ophthalmology specialization program in Poland, with the greatest impact on surgical training. Trainees providing care to patients with COVID-19 are more likely to negatively assess the impact of the pandemic on the training process. Replacing traditional training with virtual methods was positively received by residents and considered very useful, and most residents reported a desire to maintain virtual training in ophthalmology in the future.

## Introduction

Severe acute respiratory syndrome coronavirus 2 (SARS-CoV-2) causes an acute infectious disease of the respiratory system commonly known as coronavirus disease 2019 (COVID-19), which was first identified in late 2019 in Wuhan, China [1]. Rapid spread of the virus around the world resulted in a global public health threat, and COVID-19 was declared a pandemic by the World Health Organization on March 11, 2020 [2].

The first case of COVID-19 in Poland was reported on March 4, 2020, and the subsequent increase in the number of patients forced the Polish health care system to completely reorganize its work. Immediate steps were required to mobilize most health care resources to care for patients with COVID-19 and prevent the spread of the pandemic.

In accordance with the regulations outlined by the Minister of Health on March 13, 2020 [3], 19 “COVID hospitals” intended only for patients infected with SARS-CoV-2 were established in Poland, and as the disease evolved into a pandemic, more wards and temporary hospitals took over the care of patients with COVID-19. Moreover, all hospitals suspended planned admissions, limiting their services only to patients requiring immediate medical attention and those diagnosed with neoplastic diseases [4]. This situation also affected ophthalmic patients, whose treatment in Poland practically stopped for several months, except for emergencies. In 2020, 260,000 elective cataract surgeries were performed in Poland during the pandemic—a 100,000 procedure reduction when compared to 2019.

We assumed that the changes in healthcare structure and public restrictions during the pandemic caused significant difficulties in specialist training, both in terms of clinical and surgical practice and teaching. Despite the gradual restoration of routine health services in the following months of the pandemic, the principles of social distancing continue to apply. This has resulted in the introduction of virtual teaching methods and the organization of courses, trainings, and conferences in the form of on-line meetings.

There is limited research on the effects of the COVID-19 pandemic on ophthalmology residency training thus far. Although researchers from various parts of the world (Saudi Arabia, Egypt, India, Portugal, Great Britain, and Canada) have examined the issue, their studies provide data limited to specific ethnicities [5–10]. This is the first such report from the Central Eastern European country. Almost all previous reports indicate a significant and usually negative impact of the pandemic on the process of training in ophthalmology. Because some reports were published very early in the pandemic, they do not necessarily reflect its long-term impact on the education of ophthalmologists in training.

To our best knowledge, the effect of the COVID-19 pandemic on the education of ophthalmologists in training has not been investigated in Poland. We analyzed the impact of the pandemic on ophthalmology residency training in Poland during a 1-year period since the onset of the pandemic. The data from this study will fill the gaps in currently available literature on this subject. Considering research from other countries, this will enhance a wider international perspective on how ophthalmology training programs have been affected by the pandemic. This study also provides insights into the effectiveness of new virtual training methods introduced during the pandemic. Opinions on the effectiveness of e-learning can have a huge impact on shaping future methods of specialization education and may help to decide, to return to conventional training methods once the pandemic is over or to permanently include virtual teaching in education programs, and if so, to what extent.

## Materials and methods

This study was performed in accordance with the ethical standards as laid down in the 1964 Declaration of Helsinki and its later amendments or comparable ethical standards and was approved by the Bioethics Committee of the Medical University of Bialystok.

We used an anonymous questionnaire designed by all authors. The questionnaire was created on Google Forms and distributed via email, Facebook, or WhatsApp messenger. The questionnaire was available from February 15, 2021, which is exactly 11 months after COVID-19 was officially declared a pandemic in Poland. Therefore, the survey evaluates the pandemic period between March 4, 2020, and February 15, 2021. Trainees were asked to return the filled questionnaires within 1 week. We used the residents’ university and/or hospital email addresses and the official websites and social media profiles (Facebook) of the Medical University of Bialystok, Department of Ophthalmology and the Military Institute of Medicine in Warsaw. Moreover, ophthalmology residents were invited to participate in the study through a link to the survey delivered via the social media profile of one of the authors (MR), who is a national consultant and whose social media pages are often visited by Polish ophthalmology staff. The online recipients could forward the link to friends, creating a snowball effect. The personal interviews were interviewer-based questionnaires conducted in Polish to avoid any bias concerning the understanding of the questions.

The questionnaire consisted of 24 closed-ended, single-answer, multiple-choice questions. The questionnaire was distributed to both male and female ophthalmology residents regardless of the year of training. The exclusion criterion was a lack of consent for data analysis and publication. Among 127 cases, data from only one individual were excluded for this reason. Initially, a pilot study was conducted with a small group of residents (n = 12) to optimize the survey in terms of the clarity and validity of the questions, as well as the time necessary to complete the survey. The validation and reliability were assessed as good. The survey was divided into two sections. The first section collected demographic data for the respondents, such as sex, marital status, place of residence, and living arrangement (alone or with spouse, parents, children, etc.). The questions contained in the second section related to the health situation of the hospital, the organization of specialization training for residents in ophthalmology during the pandemic, and the assessment of virtual education methods.

Participation in the survey was completely anonymous and voluntary. One of the questions obtained the respondents’ consent for statistical analysis of anonymous data and their use for the purpose of scientific publications. A copy of the questionnaire is included in the additional materials (S1 Appendix).

### Statistical analysis

Statistics were calculated in R (version 3.5.1.). The variables are presented using basic descriptive statistics depending on the scale used for measurement. Nominal variables were compared between groups with the chi-square test or the Fisher’s exact test. The normality of the distribution of quantitative variables was assessed using the Shapiro-Wilk test, indicators of skewness and kurtosis, and via visual assessment of histograms. Equality of variance was assesses using the Leven test. Student’s t-test or the Mann–Whitney U-test was used to compare quantitative variables between two groups. Comparisons among three groups were made using analyses of variance (ANOVAs) with Tukey’s post hoc test or the Kruskal–Wallis test with Dunn’s post hoc test. A mean/median difference (MD) with a 95% confidence level was also calculated as appropriate. The significance level was set to p = 0.05.

## Results

Of the 126 ophthalmology residents who participated in the study, 102 (81%) were women, and 24 (19%) were men. The socio-demographic characteristics of the studied group are shown in Table 1.

**Table 1:** Sociodemographic characteristics of the studied group of trainees.

Characteristic	Total group	% of group
	n = 126	
Sex		
Female	102	81.0
Male	24	19.0
Marital status		
Single	53	42.1
Married with children	46	36.5
Married without children	27	21.4
Living with		
Family	61	48.4
Partner	36	28.6
Friends	1	0.8
Single	28	22.2
Place of residence		
Village	9	7.1
City with up to 50k citizens	6	4.8
City with 50–150k citizens	19	15.1
City with 150–500k citizens	35	27.8
City with over 500k citizens	57	45.2

In the survey, 42 (33%) residents (COVID group) reported that they had been re-assigned to work with patients with COVID-19. Among them, 31 (24.6%) treated patients with COVID-19 in the ophthalmology ward, while 11 (8.7%) worked with COVID-19 patients in other wards (infectious or emergency medical care). The non-COVID group included 84 (67%) residents working in the ophthalmology ward for patients without COVID-19. No differences in baseline characteristics were observed between these two groups.

Out of the 126 respondents, 98 (77.7%) had had a SARS-CoV-2 virus polymerase chain reaction (PCR) test, with 24 (24.5%) and 74 (75.5%) exhibiting positive and negative results, respectively. During the study period, 26 (20.6%) residents were isolated at home for an average period of 12 (10–21) days due to a positive SARS-CoV-2 virus test result and/or symptoms of COVID-19. This group included seven (16.7%) residents in the COVID group (mean isolation duration of 18 days) and 19 (22.6%) residents in the non-COVID group (mean isolation duration of 12 days) (p > 0.05). Additionally, a total of 29 (23%) residents were quarantined for a mean of 10 days (range: 7–15 days).

Total or partial restriction of elective ophthalmic surgery was reported by 117 (92.8%) residents. The above-mentioned limitations were also associated with the introduction of a new system of work, which consisted of only treating patients with urgent ophthalmic diseases that could not be postponed due to the risk of permanent deterioration of eyesight or the local condition of the eye. Total or partial limitations in the number of people in the operating theater were reported by 68 (54%) respondents (Table 2).

**Table 2:** Limitations in the operating theater during the COVID-19 pandemic as assessed by residents working and not working with patients with COVID-19.

Characteristic	Total group	COVID group	Non-COVID group	p
	n = 126 (%)	n = 42 (%)	n = 84 (%)	
Limits in planned ophthalmic surgeries				
Complete stop	11 (8.7)	6 (14.3)	5 (6.0)	0.393
Partial stop	33 (26.2)	12 (28.6)	21 (25.0)	
Partial stop with period surgery depending on the situation	73 (57.9)	21 (50.0)	52 (61.9)	
No restrictions	9 (7.2)	3 (7.1)	6 (7.1)	
Limits in number of people allowed on the operating block				
Complete	13 (10.3)	4 (9.5)	9 (10.7)	0.859
Partial	55 (43.7)	20 (47.6)	35 (41.7)	
No limits	58 (46.0)	18 (42.9)	40 (47.6)	

A total of 113 (89.7%) respondents believed that the implementation of specialized ophthalmology training was negatively impacted by the pandemic, with 79 (62.7%) and 34 (27%) respondents reporting this disruption as partial or complete, respectively. According to 112 (88.9%) respondents, the pandemic had a negative impact on the acquisition of surgical skills, with 78 (61.9%) assessing this disruption as complete and 34 (27%) assessing the disruption as partial. No significant differences in overall assessments were observed based on sex, place of residence, marital status, or provision of care to patients with COVID-19. Among trainees who were of the opinion that the pandemic negatively affected the specialization process or the acquisition of practical experience in ophthalmology, those from the COVID group and women reported a higher degree of negative impact (p = 0.008 and p = 0.015 for the specialization process, respectively; p = 0.014 and p = 0.021 for practical experience, respectively) (Tables 3 and 4).

**Table 3:** Assessment of the negative impact of the COVID-19 pandemic on the implementation of the specialization program and the acquisition of practical skills.

Characteristic	Total group	COVID group	Non-COVID group	p
Scope of the negative impact of the COVID-19 pandemic on specialist training in ophthalmology	n = 113 (%)	n = 37 (%)	n = 76 (%)	
<25%	28 (24.8)	4 (10.8)	24 (31.6)	0.008
25–50%	49 (43.4)	14 (37.8)	35 (46.0)	
50–75%	23 (20.3)	11 (29.7)	12 (15.8)	
75–100%	13 (11.5)	8 (21.6)	5 (6.6)	
Scope of the negative impact of the COVID-19 pandemic on the acquisition of practical experience in ophthalmology	n = 112 (%)	n = 38 (%)	n = 74 (%)	
<25%	16 (14.3)	3 (7.9)	13 (17.6)	0.014
25–50%	50 (44.6)	14 (36.8)	36 (48.6)	
50–75%	26 (23.2)	8 (21.1)	18 (24.3)	
75–100%	20 (17.9)	13 (34.2)	7 (9.5)	

**Table 4:** Assessment of the negative impact of the COVID-19 pandemic on the implementation of the specialization program and the acquisition of practical skills according to sex.

Characteristic	Total group	Female	Male	p
Scope of the negative impact of the COVID-19 pandemic on specialist training in ophthalmology	n = 113 (%)	n = 93 (%)	n = 20 (%)	
<25%	28 (24.8)	19 (20.5)	9 (45.0)	0.015
25–50%	49 (43.4)	40 (43.0)	9 (45.0)	
50–75%	23 (20.3)	23 (24.7)	0	
75–100%	13 (11.5)	11 (11.8)	2 (10.0)	
Scope of the negative impact of the COVID-19 pandemic on the acquisition of practical experience in ophthalmology	n = 112 (%)	n = 93 (%)	n = 19 (%)	
<25%	16 (14.3)	9 (9.7)	7 (36.8)	0.021
25–50%	50 (44.6)	44 (47.3)	6 (31.6)	
50–75%	26 (23.2)	24 (25.8)	2 (10.5)	
75–100%	20 (17.9)	16 (17.2)	4 (21.1)	

During the pandemic, 25 (19.8%) of the surveyed residents participated in traditional training or stationary courses organized under a sanitation regimen. On the other hand, 119 (94.4%) trainees participated in specialization training organized online. Among them, 118 (99.2%) residents positively assessed their substantive value and usefulness in everyday clinical practice. The most common platforms used for on-line meetings were Zoom (62.7%) and Microsoft Teams (50.8%). Ninety-nine (78.6%) respondents believed that at least some of the training courses and conferences should be maintained online once the pandemic is over. Seventeen participants (13.5%) reported that, after the pandemic ends, they would like to undergo training and specialization courses in virtual format only, while 10 (7.9%) reported that they would like to undergo training in a traditional, stationary format only. There were no statistically significant differences in responses regarding participation in virtual courses, their usefulness, or possible continuation after the pandemic based on sex, place of residence, marital status, or provision of care to patients with COVID-19. However, there was a statistically significant correlation between a positive substantive assessment of online courses/training and a positive SARS-CoV-2 test result (p = 0.022).

Among 78 residents who were involved in research activities before the pandemic, 65 (83.3%) confirmed a negative impact of the pandemic on their research work. There were no correlations between the limitation of scientific activity and sex, marital status, place of residence, provision of care to patients with COVID-19, and a positive test result.

## Discussion

The present study revealed that limiting numbers of hospital admissions and scheduled procedures in ophthalmology departments negatively impacted the implementation of the specialization program for ophthalmology residents in Poland and affected the acquisition and improvement of their surgical skills. Furthermore, the impact was perceived to be greater among residents working in ophthalmic wards dedicated to patients with COVID-19 and among female trainees. We also found that working in wards dedicated to patients with COVID-19 did not pose a higher risk of the disease among residents. Moreover, online training and courses were considered effective and rewarding forms of teaching, with most residents wanting to maintain virtual training in the future.

Our results rise a concern that, if current residents are unable to receive proper training, patients may be unable to undergo procedures that are safe and implemented in time. In addition, this may compromise the quality of training for years to come, as these residents will go on to train individuals later in their lives. However, the positive opinions regarding virtual or simulated training are very important. This is significant in that, it may expand access to training for individuals who may not always be able to participate in-person sessions for conferences, meetings, etc. This would in turn increase the availability of qualified ophthalmologists and improve patient care. Furthermore, if it is true that virtual methods are just as effective as traditional methods, this may help to increase the availability of ophthalmologists by reducing classroom time, as having virtual options that can be accessed at any time would free them to be involved in patient care and research.

Six similar studies have been conducted in various countries [5–10], and our study is the third report from Europe. The course of the pandemic has differed in each of the European countries, and our research offers insights into the situation in the eastern region of the continent.

The development of the SARS-CoV-2 pandemic and its rapid spread forced health systems in individual countries to take radical steps to cope with the increased number of patients and stem the spread of the virus. Many hospitals or wards have been reorganized and dedicated solely to the care of patients with COVID-19. Some ophthalmology residents have also been deployed to care for patients with COVID-19. In our group, 8.7% of respondents were temporarily transferred to work in infectious wards or emergency medical care during the pandemic. In Portugal, as many as 97% of the surveyed ophthalmology residents worked in hospitals assigned to treat patients infected with SARS-CoV-2, although only 25% of them worked in the so-called COVID teams [6]. Approximately 47% of COVID team residents, which constituted 12% of the entire group analyzed, continued their work in the ophthalmology department, while the rest (53%)—which constituted 13.5% of the entire group—worked in COVID-19 departments. Moreover, in other countries, many ophthalmology residents have been redeployed to work with patients with COVID-19: 39% in the UK [9], 24.6% in India [7], 18.3% in Saudi Arabia [5], and 4.9% in Canada [10]. This redeployment periodically interrupted the implementation of the specialization program in ophthalmology. In the UK, 77% of the residents who were redeployed felt that this negatively impacted their ophthalmology training [9].

Another negative effect of the pandemic was the reduction in the provision of planned health services, including a drastic reduction in the performance of planned surgical operations. The largest estimated decrease in surgical procedures and outpatient consultations among all fields of medicine was recorded in ophthalmology (79%) [11]. Since the work of ophthalmologists has been recognized as high-risk for SARS-CoV-2 infection due to close contact with patients, many national and international ophthalmological organizations and societies developed recommendations for the management of the ophthalmic patient at the beginning of the pandemic [12]. As a result, direct ophthalmological examination was significantly limited to emergency cases only. In other situations, use of telemedicine or postponement of ophthalmological visits was recommended. Elective ophthalmic surgeries, which constitute the vast majority of ophthalmic procedures, were almost totally suspended.

Based on the results of our survey, 92.8% of ophthalmic centers in Poland had suspended all elective ophthalmic surgeries, including minor ophthalmic procedures, cross-linking, and laser procedures, and training in this field was almost completely stopped. As reported in Portugal [6], only intravitreal injections for age-related macular degeneration (AMD) and diabetic maculopathy were not suspended. A drastic reduction in surgical training was also noted in other countries, ranging from 72% to 94% [5–9]. The period of their suspension depended on the current epidemiological situation of the pandemic in the country. Moreover, in more than half of cases, the number of people allowed to stay in the operating theater was reduced to the necessary minimum. This significantly limited the possibility of learning by observing the operations performed live.

At the peak of the pandemic in Portugal and the United Kingdom, restrictions affected almost 100% of scheduled operations [6, 9], while scheduled operations were drastically recued in India and Saudi Arabia [5, 7]. Cataract surgery was the most restricted form of surgery. In international studies by Ferrara et al. [13], more than half of the residents (53.1%) reported that they were unable to perform routine cataract surgery during the pandemic. Up to 99% of Portuguese residents did not participate in any cataract surgery at that time [6]. In the UK, 80% of ophthalmology residents were significantly concerned about the negative impact of the pandemic on their training in cataract surgery, given that in the UK, ophthalmology residents are expected to perform at least 350 procedures of cataract phacoemulsification by the end of training [9]. In India, 62.4% of residents reported their surgical training had deteriorated by 50% or more [7].

A survey conducted among 414 residents from around the world, in which representatives of 32 countries took part (mostly from Italy [28%], Great Britain [24%], and the Netherlands [10%]), revealed that surgical and clinical activity in ophthalmology decreased by 52.6% [13]. In this previous study, 74.6% of the trainees reported that their clinical and surgical practice decreased by more than 75% when compared to the pre-pandemic state, and the average number of surgeries performed in the previous 2 months was 2.2.

In many places, a rotational work cycle was introduced, and medical teams that worked in shifts were formed to avoid cross-contamination. These changes in the work. In our survey, 89.7% of the respondents said the pandemic negatively affected their ophthalmology training, which is similar rates reported in other studies (81–93.8%) [5–7, 9].

In our study, among trainees who reported a negatively impact of the pandemic on the ability to gain practical experience in ophthalmology, residents providing care to patients with COVID-19 reported a much higher degree of this negative impact. This seems perfectly understandable considering that, due to the changing nature of work for these residents, their ophthalmic training program has been drastically interrupted or limited.

On the other hand, the pandemic has not appeared to significantly affect the acquisition of theoretical knowledge. In Indian studies, 47.2% of residents believed that the SARS-CoV-2 pandemic had a negative impact on theoretical learning, but 65% of this group believed that the extent of the impact was less than 50% [7]. Only around 15% of trainees in the UK reported being worried about the lack of classroom teaching [9]. Among Canadian ophthalmology residents, 43% were worried about losing clinical skills, although significantly fewer were concerned about their surgical skills deteriorating (55%) [10].

An international survey by Ferrara et al. [13] showed that 34% of ophthalmology residents from different countries believed that the development of the pandemic caused irreversible shortages in their specialization training, and another 36% were uncertain in this regard but do not rule it out. In their study, up to 52% of respondents believed that the measures taken by their institutions to maintain the effectiveness of specialized training were not appropriate or sufficient [13].

Epidemic restrictions significantly reduced the traditional stationary forms of training and participation in scientific conferences. This led to the introduction of new virtual forms of teaching, such as wetlabs, virtual simulators, webinars, podcasts, vodcasts, online courses, and symposia, the usefulness of which has already been confirmed [14–16]. As shown by the international research of Chatziralli et al. [17] during the pandemic, use of online training increased significantly, and Zoom turned out to be the most popular platform used for this purpose.

In our study, up to 99.2% of participants positively assessed the substantive value and usefulness of virtual forms of training in everyday clinical practice. Similarly, 88% of British residents and 75.5% of trainee ophthalmologists in India found virtual training methods useful [7, 9]. Among young Egyptian ophthalmologists, almost 61% found online activity very educational, and a further 25% found it moderately beneficial [8].

A separate issue is the usefulness of virtual forms of training in teaching surgical skills. The pandemic suddenly increased the need for virtual surgical simulators [18]. Currently available cataract surgery simulators include Eyesi (VR Magic Holding MicroVisTouch ™ (ImmersiveTouch, Chicago, IL, USA) and the HelpMeSee (HMS) Eye Surgey Simulator (HelpMeSee Inc. NY, USA) [19]. Szigiato et al. [10] reported that 19.6% of respondents had access to a surgical simulator (Eyesi) and that 18.6% practiced using wetlabs [13]. In their study, 88.6% of respondents found simulator training very helpful in maintaining or improving surgical skills. Assessments of the usefulness of simulator training among young Egyptian ophthalmologists revealed a high or very high level of satisfaction with this form of teaching among 59.8% of respondents and an average degree of satisfaction among most others (33.8% of respondents) [8]. If the lockdown caused by the pandemic were to be prolonged, most of the Egyptian trainees reported that they would want traditional surgical training to be replaced by simulator-based training (66.7%), video sessions (48.7%), or wetlabs (41%) [8].

Although research is not a part of traditional specialization training programs in ophthalmology, many residents partake in research. Among trainees involved in scientific work before the pandemic, over 83% of respondents in our study confirmed the negative impact of the pandemic on scientific activity. Only Silva et al. investigated the impact of the pandemic on scientific work [6], reporting that 43% of Portuguese ophthalmology residents continued their research activities during the pandemic. In their study, as many as 80% of respondents said that they had more time for such activities than in the period before the pandemic. Indeed, 32% of Portuguese residents engaged in research work increased the number of papers sent for publication. The difference between the impact of the pandemic on the research activities of Polish and Portuguese residents is probably due to the different course of the pandemic in these countries, which was more intense in Portugal. In addition, our research covered the 11-month period of the pandemic in Poland. Epidemiological restrictions were relaxed after the initial almost complete lockdown from March to May 2020, and the former work system was largely reinstated in the summer months. The Portuguese research covers the very early period of the pandemic and ends in April 2020, which is the period during which constraints were greatest.

This study is not free from limitations. The very nature of survey methodology shows that such studies are not validated prior to its applications. Moreover, the self-administered survey questions are not standardized, and the changing pandemic scenario means that every now and then new issues appear that were not previously covered in the survey. Before conducting the survey, we sent the questionnaire to a small number of residents for optimization, although we realized that it is only possible to objectively assess the value of a given survey and notice its possible shortcomings after the survey has been completed. It should also be emphasized that the survey is based only on the subjective assessment of the respondents, and not on objective data. On the other hand, given that the feelings and opinions of the surveyed ophthalmology residents are the most important aspects of this study, survey methods appear to reflect the appropriate approach. The strength of our work is that we investigated the situation among ophthalmology residents during the 11 months of the pandemic, during which there were periods of complete lockdown and significant easing of epidemiological restrictions. In contrast, most other studies investigated the period from April to May of 2020, when there were the most restrictions. Another limitation may be that our study represents data from only one country. On the other hand, such material is homogeneous and relates to a specific epidemiological situation in a particular country. Similar research from other countries may provide information on how the pandemic affects the delivery of the training programs depending on the country’s pandemic strategy.

## Conclusions

The impact of the pandemic on education among ophthalmologists may raise the question of whether trainees who complete the 5-year specialization period will be sufficiently prepared to work with patients. Until the end of the pandemic, efforts should be made to ensure that the education of residents is as effective as possible despite pandemic-related limitations to prevent a potential negative impact on the care provided to ophthalmology patients.

Based on our conclusions, we formulated the following recommendations to ensure the quality of ophthalmic specialist training during the pandemic:

1. Intensifying surgical skill training from the first year of residency
2. Introducing virtual surgical simulators (i.e., cataract surgery simulators) to all ophthalmology departments to develop practical skills among trainees
3. Providing additional surgical training for residents redeployed to COVID-19 units during the pandemic
4. Introducing and improving virtual forms of teaching on a large-scale, including wetlabs, virtual simulators, webinars, podcasts, vodcasts, online courses, and symposia.

## Supporting information

S1 Appendix The questionnaire survey. (DOCX)
