# Supplementary material for: Impact of the COVID-19 pandemic on ophthalmic specialist training in Poland
Source: PLoS One. 2021 Sep 24;16(9):e0257876. doi: 10.1371/journal.pone.0257876 (PMC8462680; doi:10.1371/journal.pone.0257876)
Supplement: S1 Appendix — (DOCX) [file pone.0257876.s001.docx]

**Survey for ophthalmology residents:**

**”The evaluation concerning the influence of SARS-CoV2 pandemic on the realisation of the special training programme among ophthalmology residents.”**

The aim of the survey was to evaluate the influence posed by SARS-CoV2 pandemic on the realisation of the special training programme in the field of ophthalmology, as well as mental and physical health among ophthalmology residents. The survey was completely anonymous, and the members of the research team shall observe complete confidentiality and use the data obtained from the survey solely for the purpose of elaborating studies. Each respondent has the right to withdraw from the study at every stage of its duration. Filling the entire survey signifies an adhesion to participation in scientific research.

DEMOGRAPHIC DATA:

**1. Sex**

□ Female □ Male

**2. Marital status:**

□ Single □ Married with kids □ Married without kids

**3. Living with:**

□ Family □ Friends □ Partner □ Alone

**4. Place of residence:**

□ Village □ City up to 50k residents □ City up to 50-150k residents □ City up to 150-500k residents □ City >500k residents

**5. What department are you working in?**

□ Dealing only with Covid-19 patients

□ Dealing partially (temporarily) with Covid-19 patients

□ Not dealing only with Covid-19 patients

**6. Have the hospital guidelines on admitting patients and performing surgeries during the COVID19** **pandemic been introduced**

□ Yes, immediately

□ Yes, gradually as the pandemic develops

□ Only partially

□ No

**7.** **Have planned ophthalmic surgeries been limited?**

□ completely

□ partially, from the start of the pandemic to the present time

□ partially, but only periodically, depending on the situation

□ not limited

**8. Was the number of staff allowed in the operating theater limited during the pandemic?:**

□ completely

□ partially

□ not limited

**9. Have you been tested for COVID-19?**

□ No

□ Yes, it was negative

□ Yes, I am waiting for the result

□ Yes, it was positive

**10. Were you in quarantine during the Covid-19 pandemic?**

□ No

□ Yes, once

□ Yes, more than 1 time (how many? .........)

**11. If you were in quarantine, how many days did it last?**

………………. Days

**12. Were you in isolation (release) because of Covid-19 infection?**

□ No

□ Yes, once

□ Yes, more than 1 time (how many? .........)

**13. If you were on release (sick leave), how many days did it last**?

………………. Days

**14. During the Covid-19 pandemic, did you participate in courses, trainings or conventions that were conducted in a stationary manner?**

□ Yes

□ No

**15. During the Covid-19 pandemic, did you participate in courses, trainings or meetings that were conducted remotely on-line?**

□ Yes

□ No

**16. Which applications were used for on-line meetings?**

□ Zoom platform

□ Microsoft Teams

□ Google Hangouts

□ Adobe Connect

□ Other:…

□ I don't know

**17. Were the organized courses, meetings, on-line training / e-learning meetings valuable in your opinion?**

□ Yes

□ Only partially

□ No

□ I have no opinion

**18. Do you think that after the pandemic is over, training and conferences in the form of on-line and e-learning should be continued?**

□ Yes, online only

□ Partly on-line, partly stationary

□ No

□ I have no opinion

**19. In your opinion, did the Covid-19 pandemic have had a negative impact on the implementation of the ophthalmology specialization program?**

□ Yes, absolutely

□ Partially yes

□ No

□ I have no opinion

**20. If the impact was negative, to what extent?**

□ <25% □ 25-50% □ 50-75% □> 75%

**21. Do you think the pandemic has had a negative impact on the acquisition of practical skills in ophthalmology?**

□ Yes

□ Partially

□ No

□ I have no opinion

**22. If the impact was negative, to what extent?**

□ <25% □ 25-50% □ 50-75% □> 75%

**23. Was your research activity limited due to the pandemic?**

□ Yes

□ Only partially

□ No

□ I am not involved in any research activities
